# Ionic liquid flow along the carbon nanotube with DC electric field

**DOI:** 10.1038/srep11799

**Published:** 2015-07-02

**Authors:** Jung Hwal Shin, Geon Hwee Kim, Intae Kim, Hyungkook Jeon, Taechang An, Geunbae Lim

**Affiliations:** 1 Department of Mechanical Engineering, Pohang University of Science and Technology (POSTECH), San31, Hyoja-dong, Pohang, Gyungbuk, 790-784, Republic of Korea; 2 Department of Mechanical Design Engineering, Andong National University, Andong, Gyungbuk, 760-749, Republic of Korea

## Abstract

Liquid pumping can occur along the outer surface of an electrode under a DC electric field. For biological applications, a better understanding of the ionic solution pumping mechanism is required. Here, we fabricated CNT wire electrodes (CWEs) and tungsten wire electrodes (TWEs) of various diameters to assess an ionic solution pumping. A DC electric field created by a bias of several volts pumped the ionic solution in the direction of the negatively biased electrode. The resulting electro-osmotic flow was attributed to the movement of an electric double layer near the electrode, and the flow rates along the CWEs were on the order of picoliters per minute. According to electric field analysis, the *z*-directional electric field around the meniscus of the small electrode was more concentrated than that of the larger electrode. Thus, the pumping effect increased as the electrode diameter decreased. Interestingly in CWEs, the initiating voltage for liquid pumping did not change with increasing diameter, up to 20 μm. We classified into three pumping zones, according to the initiating voltage and faradaic reaction. Liquid pumping using the CWEs could provide a new method for biological studies with adoptable flow rates and a larger ‘Recommended pumping zone’.

The manipulation of droplets or liquids is important in various applications such as printing or patterning^1^,^2^, biological assays^3^,^4^, and chemical reactions^5^,^6^. Channels^7^,^8^, nozzles^9^,^10^,^11^, or tubes^12^,^13^,^14^ are commonly used to guide fluids, and the transported liquids are usually enclosed and restricted by solids. These closed systems present several challenges, such as a high flow resistance and frequent clogging. Recently, open systems in which the liquid moves along the outer surface of a solid, have been introduced in the form of flexible fiber arrays^15^, rigid nanowires^16^, spider silks^8^, cactus^17^, and conical copper wires^18^.

Transported droplets or liquids can be controlled using a variety of approaches, including architectural tapering with conical fibers^18^,^19^,^20^ or voltage application^21^,^22^,^23^. The former relies on the self-propelled behavior of the liquid along the conical fibers, driven by a surface physiochemical gradient, with droplets balanced at particular positions. The self-propelled mechanism has been used to harvest water from humid air^24^ or to separate micro-sized oil droplets from water^25^. Transported liquid or continuous liquid flow can occur along rigid nanowires via DC electric field application; an example of this is iontophoretic delivery^26^.

Liquid pumping of dielectric liquids along the outer surfaces of electrodes was first introduced by Faraday *et al.* in 1839, under tens of kilovolts biasing^27^. Further advancements by Sumoto (1956)^28^ and Daba (1971) showed that the pumping effect could be enhanced by increasing the applied voltage and decreasing the electrode diameter^29^. Recently, a study has demonstrated ionic liquid flow along rigid nanowires under the application of small voltages (on the order of 4 V) for electrodes with diameters of hundreds of nanometers^16^. However, for biological studies, a better understanding of the ionic solution pumping mechanism is required, with a focus on the amount of liquid that can be transported, in addition to the faradaic reaction and water electrolysis.

In this study, we fabricated CNT wire electrodes (CWEs) and sharpened tungsten wire electrodes (TWEs) having various diameters to study the liquid pumping of an ionic solution. The electric field between two electrodes was analyzed by COMSOL and the flow rate of the transported liquid was assessed by changing the applied voltage and distance between the two electrodes. The ionic solution pumping mechanism along the electrode was described, based on our experimental results and analysis.

## Experimental set-up

**Figure 1:**
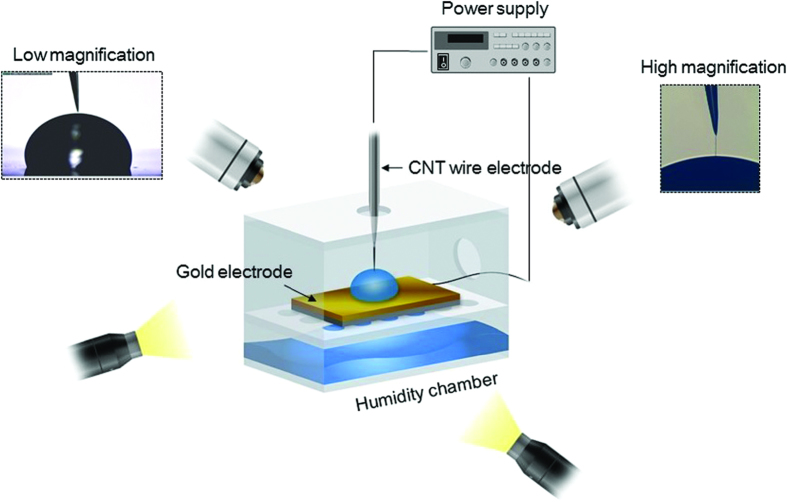
*Experimental* set-up. A carbon nanotube wire electrode (CWE) and a gold electrode were placed in a humidity chamber (>85% relative humidity (RH)). Two optical microscopes and light sources were used to examine the electrodes. The chamber and the CWE were precisely controlled by means of three-axis stages, and a DC electric field was applied between the two electrodes.

Figure 1 shows the experimental set-up, which consisted of a chamber, a CWE, a gold electrode, three-axis stages, two light sources, two optical microscopes, and a power supply. The chamber was divided into two parts. The bottom part contained wet tissues, and the intermediate layer had many holes to retain a relative humidity of >85% in the chamber; these conditions prevented evaporation of the liquid droplet on the gold electrode and facilitated liquid transport along the CWE. The gold electrode was placed in the chamber’s intermediate layer, and a 2 μL liquid droplet was placed on the gold electrode. The chamber and the CWE were connected separately to a three-axis stage to allow independent control of the electrode placement, as well as placement for optical imaging with the microscopes. The power supply applied a DC voltage between the CWE and the gold electrode.

### Fabrication of CWEs and sharpened TWEs^30^,^31^

Sharpened TWEs were fabricated using a dynamic electrochemical etching. A 300 μm diameter TWE and a Pt electrode were immersed in a NaOH electrolytic solution. An electric potential of 7 V was applied between the TWE and Pt electrode. The TWE was moved up and down slowly to establish smooth morphology.

**Figure 2:**
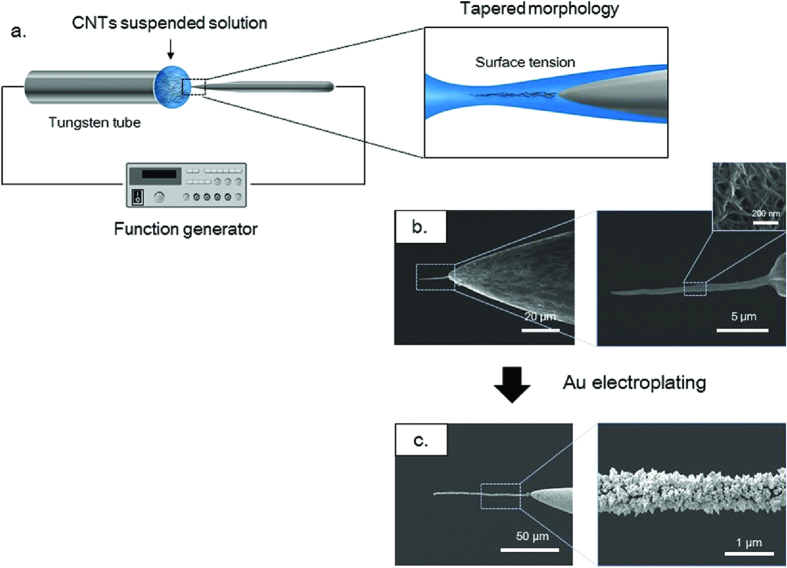
CWE fabrication process. (**a**) Schematic diagram of CWE fabrication. (**b**) Scanning electron microscopy (SEM) images of the CWE. The inset shows the individual CNTs. (**c**) SEM images of the CWE after Au electroplating.

CWEs were fabricated on sharpened TWEs using dielectrophoresis (DEP) and surface tension. A TWE was submerged in a solution, containing CNTs. An AC electric field was applied between the tungsten tube and the TWE (Fig. 2a). The CNTs in the solution were attracted to the TWE by dielectrophoresis (DEP) force. The collected CNTs were compressed by surface tension during solution evaporation. The fabricated CWEs naturally have a tapered architecture, due to the meniscus on the TWE (inset of Fig. 2a). The applied voltage and frequency were controlled to achieve the desired CNW diameter, given the tungsten morphology. The CWE diameter could be increased up to several tens of microns by repeating this process. Figure 2b shows a fabricated CWE; the CWE consisted of many individual CNTs.

Generally in this method, the CNTs of the CWE were combined by van der Waals force. When a DC electric field was applied to the immersed CWE, the gathered CNTs could be re-dispersed. To strengthen the bonding force between the CNTs, gold nanoparticles were coated on to CWEs by electrodeposition. A gold solution of 5 mM HAuCl_4_ · 4H_2_O and 500 mM HBO_3_ was introduced by means of a tungsten tube. The fabricated CWE was submerged in the gold solution. A DC electric bias of 1 V was applied between the two electrodes for 20 sec. Figure 2c shows the CWE coated by gold nanoparticles and the diameter was about 800 nm diameter.

### Liquid pumping phenomenon under DC conditions

**Figure 3:**
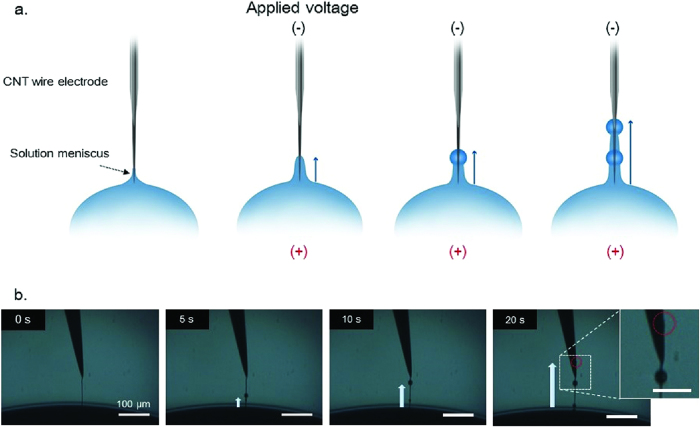
Liquid pumping of an ionic solution along a *CWE.* (**a**) Schematic diagram of the ionic solution transport along the *CWE*. (**b**) Optical time-lapse images of the ionic solution transported along the *CWE* over 20 sec period.

The CWE was manipulated by a three-axis stage and placed into an ionic solution droplet. A KCl ionic solution was used in this study because K^+^ and Cl^–^ ions have similar electric mobility. Figure 3a shows that the liquid transported liquid along the CWE when a DC electric field was applied; note that the thin liquid ‘precursor film’ was excluded. When the CWE was submerged, a meniscus formed along the CWE. Without an external force, the liquid spread along the CWE due to its tapered architecture and hydrophilic characteristics. This phenomenon is described in detail in Figure S1. A negative bias was applied to the CWE, resulting in pumping of the liquid along the CWE surface. During liquid pumping, some of the collected liquid generated beads along the CWE. If a DC voltage was applied between the two electrodes continuously, then the liquid flowed continuously along the CWE.

Figure 3b shows time-lapse optical microscopy images of the ionic solution pumping. In a previous study, transmission electron microscopy images (TEM) indicated the formation of a precursor film on the nanowire surface; the film thickness was ~1–10 nm. Over time, the beads on the CWE became larger (up to several tens of microns) at certain positions; some of these beads crept along the CWE.

### Electric field around the meniscus and the electro-osmotic flow along a CWE

The fact that dielectric liquids can be pumped by a DC electric potential has been known for at least 100 years^27^. The climbing of dielectric liquids along the electrodes is caused by a DEP force^29^ or an electrohydrodynamic (EHD) effect^32^. This phenomenon provides the basis for ‘water bridge’ studies^33^. However, it has been difficult to apply this phenomenon to biological studies, due to the high electric potential required, as well as the various adverse products generated by the process.

**Figure 4:**
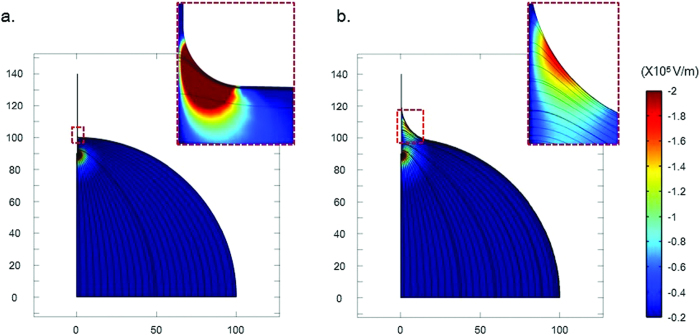
Electric field as a function of the radius of curvature (ROC) of the meniscus. Condition - droplet radius: 100 μm; length and radius of the electrode: 50 μm and 400 nm; voltage and distance: 5 V and 140 μm; ROC of the meniscus: (**a**) 1 μm and (**b**) 20 μm; streamline: electric field, surface: electric field, z-component.

From Laplace’s theorem, 1/*R*_*1*_ + 1/*R*_*2*_ = *C* = *Δp*/*ϒ*, the meniscus profile can be expressed as [*z*(*x*) = *b*·cosh(*x*/*b*)] when gravity is neglected. *R*_*1*_ and *R*_*2*_ represent the radii of curvature of the surfaces, *Δp* is the pressure difference, *b* is the electrode diameter, and *ϒ* is the surface tension. According to the above equation, the smaller the electrode diameter, the smaller and sharper the meniscus surrounding the electrode. In this study, we numerically analyzed the electric field as a function of the radius of curvature (ROC) of the meniscus, using COMSOL software. To simplify our model, the presence of an electric double layer (EDL) around the electrode was neglected, and the electrode was assumed to be a perfect conductor. An electric potential of 5 V was applied to the entire surface of the electrode, and the electrical ground condition was applied to the bottom of the droplet. Figure 4 shows the electric filed configuration and the magnitude of the *z*-directional electric field in the droplet. The *z*-directional electric field exhibited considerable strength around the meniscus and at the electrode tip. For a ROC of 1 μm (Fig. 4a), the *z*-directional electric field around the meniscus was more concentrated, due to the small profile of the meniscus compared with that for a ROC 20 μm (Fig. 4b). Thus, the smaller the electrode diameter, the more concentrated the *z*-directional electric field around the meniscus.

**Figure 5:**
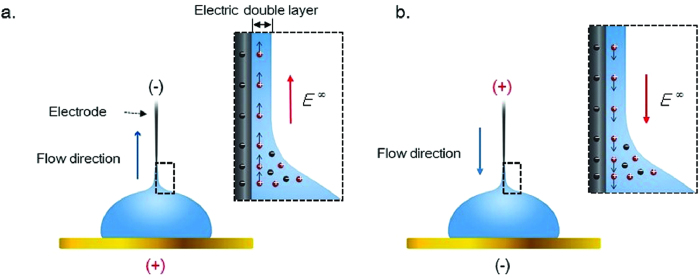
Electro-osmotic flow (EOF) by a DC electric potential. (**a**) Positive ions in the ionic solution gathered at the top electrode having a negative surface charge and formed an electric double layer (EDL). When the top electrode had a negative bias, the EDL moved upward. (**b**) When the top electrode had a positive bias, the EDL moved downward.

In our results, when the CWE had a negative bias, the liquid flowed towards the CWE (Fig. 5a). Conversely, when a positive bias was applied to the CWE, the transported liquid moved towards the gold electrode (Fig. 5b). As a result, the liquid pumping was one-directional, and the liquid flow was directed towards the negatively biased electrode with a negative surface charge. The inset of Fig. 5 shows the liquid pumping mechanism of the ionic solution. In this study, the CWEs coated by gold nanoparticles had a negative surface charge. Positive ions in an ionic solution may gather at the CWE having a negative surface charge, forming a thin EDL. The *z*-directional electric field generated around the meniscus, shown in Fig. 4, induced the mobile ion layer to move via the resulting Coulomb force. The resulting flow is the electro-osmotic flow (EOF).

### Liquid pumping with variation in the electrode diameter

The current response between a CWE or a TWE and the gold electrode was measured using a modulab system (Solartron Instruments, Elmsford, NY, USA). A scan rate of 100 mVs^−1^ was used over the voltage range of −4 V and 0 V, beginning at the open-circuit potential and sweeping to a negative bias. When a DC bias was applied between the immersed electrodes, a faradaic reaction was generated, as well as water electrolysis, upon application of higher voltages. Water electrolysis produces new products, such as hydrogen or oxygen gas bubbles at the electrodes; these products could adversely affect surrounding biological matter, such as cells or proteins. Corresponding electrochemical reactions on the anode (gold) and cathode (CWEs) proceed according to the following equations:


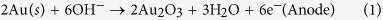



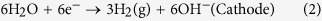


**Figure 6:**
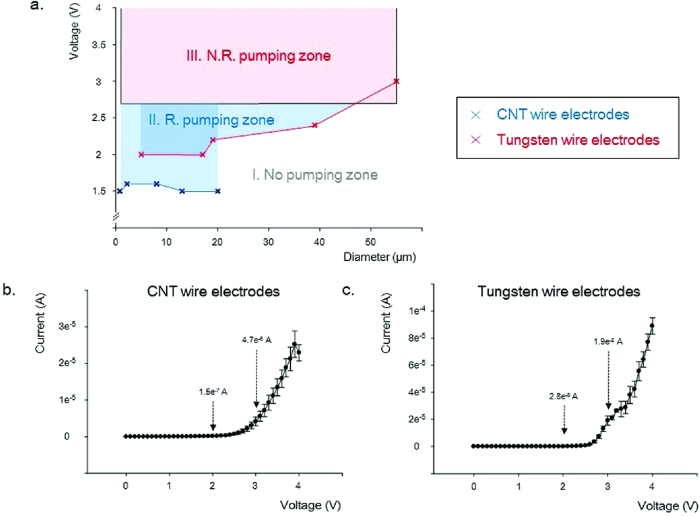
Liquid pumping of an ionic solution according to electrode diameter. (**a**) The initi**a**ting voltage for liquid pumping of a 50 mM KCl as a function of the CWE and tungsten wire electrode (TWE) diameters (*CWE* diameters: 0.8, 2, 8, 13, 20 μm; TWE diameters: 5, 17, 19, 39, 55 μm). (**b**) Current-voltage (*I-V*) response of *CWE*s immersed in a 50 mM KCl (n = 5, mean ± standard error). (**c**) *I-V* response of TWEs immersed in 50 mm KCl (n = 5, mean ± standard error).

A faradaic reaction of the CWEs and TWEs was generated above 2.7 V, and the current of the TWEs increased sharply compared with that of the CWEs (Fig. 6b,c). To assess water electrolysis, two electrodes were immersed in 50 mM KCl solution and observed under an optical microscope during DC electric field application. Above 3.9 and 3 V, bubbles were generated on the CWEs and TWEs, respectively.

As described in the electric field analysis, the *z*-directional electric field around the meniscus of small electrode was more concentrated than that of the larger electrode. Figure 6a shows the initiating voltage for liquid pumping as a function of the CWE and TWE diameters. The diameters of the CWEs and TWEs ranged from 0.8 to 20 μm and from 5 to 55 μm, respectively. As expected from the electric field analysis for TWEs, the initiating voltage for liquid pumping gradually increased with increasing diameter. However, for the CWEs, the initiating voltage for liquid pumping was similar over the entire CWE diameter range (from 0.8 to 20 μm). Because it can be difficult to fabricate CWEs larger than 20 μm in diameter, we fabricated a CNT nanosheet (size: 25 × 5 mm) composed of many individual CNTs (Fig. S2). Small droplets were gradually generated near the mother droplet from the DC electric field at ~1.5 V. Over time, the liquid crept along the CNT nanosheet facing the electrode having a negative bias. Thus, above 1.5 V, the ionic solution could be pumped along the CWE composed of many individual CNTs, regardless of the CWE diameter.

The faradaic reactions of CWEs and TWEs were generated over 2.7 V, and hydrogen bubbles were evident in optical microscopy imaging at ~3.9 and 3 V for the CWEs and TWEs, respectively. During the faradaic reaction, the liquid pumping was continuous. The CWEs and TWEs having various diameters were classified into three zones: a ‘No pumping zone’, ‘Recommended (R.) pumping zone’, and ‘Not recommended (N.R.) pumping zone’, according to the initial voltage and faradaic reaction (Fig. 6a). In the ‘No pumping zone’, liquid pumping did not occur. In the ‘R. pumping zone’, liquid pumping occurred with no faradaic reaction. Liquid pumping and the faradaic reaction occurred together in the ‘N. R. pumping zone’. For example, the TWE having a 55 μm diameter was pumped by a DC bias exceeding 3 V; however, the pumping was accompanied by a faradaic reaction. Therefore, for liquid pumping to be applied to biological studies, the voltage should coincide with the ‘R. pumping zone’ to avoid the faradaic reaction. The liquid pumping of CWEs could be initiated at lower voltage than that of TWEs, and importantly the CWEs could support a larger ‘R. pumping zone’ than that of the TWEs.

### Flow rates of transported liquids

In this study, flow rates associated with 800 nm diameter CWEs were evaluated over a voltage bias range of 1.5 to 3.5 V. When a droplet is generated at a certain position on the CWE, the droplet increased in size at this position or crept along the CWE. Note that when the droplet size increased, we assumed that the transported liquid was used to increase the droplet volume. The flow rates of the transported liquids were analyzed for two conditions: the first condition corresponded to a change in the bias voltage while the distance between the electrodes remained fixed, and the second condition involved a change in the separation distance of the electrodes for a fixed bias.

**Figure 7:**
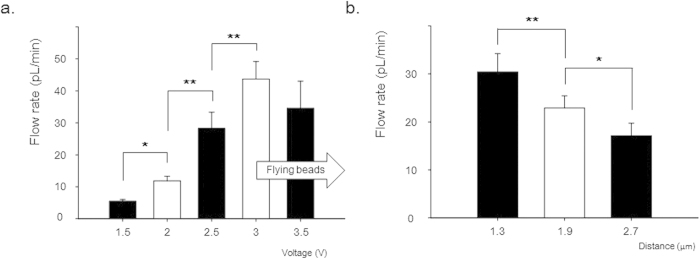
Flow rates of liquid pumped along the *CWEs*. (**a**) The flow rates increased with the applied voltage, with the exception of an applied voltage of 3.5 V condition (50 mM KCl; droplet volume: 2 μL).(**b**) The flow rates decreased with increasing distance between the two electrodes (applied voltage: 2.5 V; 50 mM KCl). Values were analyzed by paired t-test, mean ± standard error, n = 10, *p < 0.05, **p < 0.01.

For the first condition, the applied voltage was varied from 1.5 V to 3.5 V, while the other conditions remained fixed (Fig. 7a). The Helmholtz-Smoluchowski slip velocity (~*εξE*/*η*) describes the EOF, in which *ε* is the dielectric constant, *ξ* is the zeta potential, *E* is the applied electric field, and *η* is the solution viscosity. According to this equation, the flow rate of transported liquid is linearly proportional to the applied voltage, and inversely proportional to the distance between the two electrodes. In our results, the flow rates of the pumped liquid along the CWE increased with increasing applied voltage, with the exception of the 3.5 V bias. At 3.5 V, the liquid was pumped along the CWE, and small beads were expelled from the mother droplet. This phenomenon is caused by the repulsive force in the ionic solution close to the electrode^34^. These flying beads from the transported liquid were induced by the electric field and reduced the amount of transported liquid along the CWE. For this reason, the flow rate under a 3.5 V bias decreased, compared with that for a 3 V bias. For a bias of 1.3 to 3 V, the flow rates were linearly proportional to the applied voltages (*R*^2^ = 0.97).

In the second part of the experiment, the distance between the two electrodes was changed, while the other conditions remained fixed (Fig. 7b). The distance was changed by varying the volume of the mother droplet; the depth of the CWE dip into the mother droplet was fixed. The flow rates of the pumped liquid along the CWE decreased with increasing distance between the two electrodes. From 1.3 to 2.7 μm separation, the flow rates were inversely proportional to the distance between the two electrodes (*R*^2^ = 0.97). These two results demonstrate that the EOF played a significant role in liquid pumping along the CWE by a DC electric field. The flow rate using the CWEs was on the order of picoliters per minute; this range is applicable to various biological studies, including single cells (D ~ 10 μm; V ~ 0.5 pL).

## Conclusions

In summary, CWEs and sharpened TWEs having various diameters were fabricated to study liquid pumping of an ionic solution. When a DC electric field was applied between two electrodes dipped into an ionic solution, liquid was pumped along the electrode having a negative bias. During liquid pumping, the liquid gathered and formed beads at several positions along the electrode. The liquid pumping of an ionic solution is caused by the movement of mobile ions (EDL) gathered around the electrode, and the resulting flow is the EOF. According to Laplace’s theorem, the smaller the electrode diameter, the smaller and sharper the meniscus around the electrode. Upon analysis of the electric field by COMSOL, the *z*-directional electric field around the meniscus of the small electrode was found to be more concentrated than that of the larger electrode. Therefore, liquid pumping with a smaller electrode can be initiated at lower voltages. Interestingly in CWEs, the initiating voltage for liquid pumping did not change, up to 20 μm diameter, because the liquid crept along the individual CNT surfaces. Regarding the initiating voltage and faradaic reaction, the pumping zone could be classified into three zones: a ‘No pumping zone’, ‘R. pumping zone’, and ‘N.R pumping zone’. The liquid pumping of an ionic solution for biological studies should be applied within the ‘R. pumping zone’ to prevent faradaic reactions. Similar to the Helmholtz-Smoluchowski slip velocity, the flow rate of the transported liquid was linearly proportional to the applied voltage, and inversely proportional to the distance between the two electrodes. The flow rates using the CWEs were on the order of picoliters per minute. We anticipate that the CWEs could provide new devices for biological studies to manipulate liquid with adaptable flow rates.

## Additional Information

**How to cite this article**: Jung Hwal Shin *et al.* Ionic liquid flow along the carbon nanotube with DC electric field. *Sci. Rep.* **5**, 11799; doi: 10.1038/srep11799 (2015).

## Supplementary Material

Supplementary Information
