# Corrigendum: Ionic liquid flow along the carbon nanotube with DC electric field

**DOI:** 10.1038/srep13325

**Published:** 2015-09-18

**Authors:** Jung Hwal Shin, Geon Hwee Kim, Intae Kim, Hyungkook Jeon, Taechang An, Geunbae Lim

Scientific Reports 5: Article number: 11799; 10.1038/srep11799 published online: 07022015; updated: 09182015.

This article contains errors in the Acknowledgments section.

“This work was supported by the National Research Foundation of Korea (NRF) grant funded by the Korea government (MEST) (No.2012R1A1A2007580).”

should read:

“This work (2012R1A2A2A06047424) was supported by Mid-career Researcher Program through NRF grant funded by the MSIP. This work was supported by the National Research Foundation of Korea (NRF) grant funded by the Korea government (MSIP) (No. 2011-0030075).”

