# Supplementary material for: Ionic liquid flow along the carbon nanotube with DC electric field
Source: Sci Rep. 2015 Jul 2;5:11799. doi: 10.1038/srep11799 (PMC4488867; doi:10.1038/srep11799)
Supplement: Supplementary Information [file srep11799-s1.doc]

**Ionic liquid flow along the carbon nanotube with electric field**

**Jung Hwal Shin^1^,** Geon Hwee Kim**^1^, Intae Kim^1^, Hyungkook Jeon ^1^, Taechang An^2, *^, Geunbae Lim^1, *^**


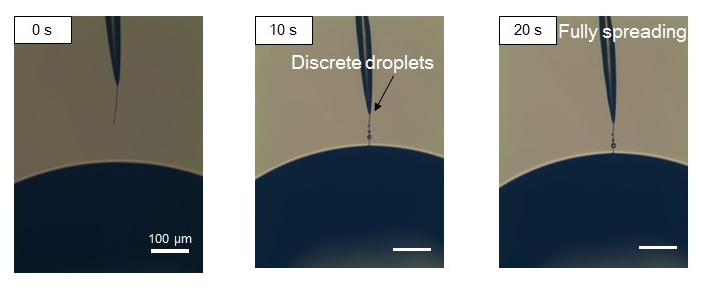


Figure S1. Liquid spreading along carbon nanotube wire electrodes (CWEs) in the absence of an external force.

The liquid droplets could be self-balanced on an anisotropic multiscale-structured conical copper wire (SCCW), depending on the apex and tilt angles of the SCCW. Figure S1 shows optical time-lapse images for a 20 sec period for an immersed CWE in the absence of an external force. Over time, several liquid droplets self-balanced at certain positions on the CWE. It is noteworthy that the fabricated CWEs had a tapered architecture; additionally, CNTs were dispersed under an oxidizing reaction upon sonication in a strong acid, resulting in hydrophilic characteristics. We attributed the self-balancing behavior of the droplets on the CWE to the tapered architecture and the hydrophilic characteristic of the CWEs.

In previous studies, the efficiency *η* (= *V_1_*/*V_2_*, where *V_1_* is the liquid droplet volume and *V_2_* is the SCCW volume encapsulated by the liquid) was used to evaluate the efficiency of the liquid manipulated by the SCCW. The SCCW exhibited a superior ability to manipulate liquid droplets at high volumes. Specifically, a SCCW having a 4.2° apex angle (smallest angle) without tilt can store more than 350-fold its own volume of liquid (*η_max_* = 361). In the CWEs, a droplet volume was calculated as *V_1_* ~ (4/3) *πR*^3^, while an encapsulated CWE volume was calculated as *V_2_* ~ *πR*^2^*h*. When the diameters of the droplet and the CWE were 15 μm and 800 nm, respectively, the efficiency was ~234 (*V_1_* ~ 1.8 pL, *V_2_* ~ 7.5 fL). This value was similar to the SCCW having an apex angle of 5.5° without tilt.


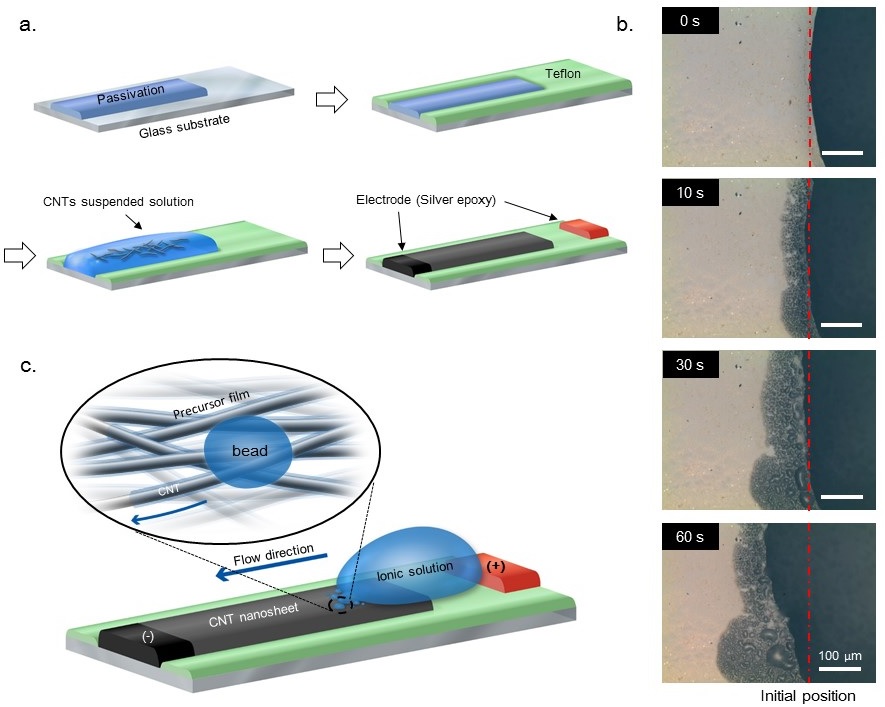


Figure S2. Liquid pumping along a CNT nanosheet.

1. Schematic diagram of fabrication of a CNT nanosheet of 25 × 5 mm dimensions.
2. Optical time-lapse images of liquid transported along a CNT nanosheet over a 60 sec period by a DC electric field of 3 V.
3. Schematic diagram of liquid transported along a CNT nanosheet.

A CNT nanosheet was fabricated on a glass substrate (size: 75 × 25 mm^2^) using evaporation deposition. Figure S2a shows the fabrication process of the CNT nanosheet. First, scotch tape was attached to the glass substrate as a passivation layer. A 2 % polytetrafluoroethylene (PTFE) solution (Teflon AF 601S1-100-6, DuPont, USA) was diluted in FC-75 (Acros Organics, Belgium), deposited onto the glass substrate, and dried at room temperature. The scotch tape was then removed from the glass substrate. A CNT-suspended solution was dispensed on the substrate surface. The substrate was placed in a 65 °C oven for 10 min to form a carbon nanosheet. Finally, a silver epoxy coated the edges of the glass substrate to create electrodes.

A 50 mM KCl solution was placed on the CNT nanosheet. A DC voltage of 1.5 V was applied between the two electrodes. Small droplets gradually formed near the mother droplet. Over time, the mother droplet crept along the CNT nanosheet, in the direction of the electrode having a negative bias. Figure S2c shows a schematic diagram of this phenomenon. Small droplets were generated from the mother droplet along the individual CNTs. A small droplet was formed when a creeping thin liquid film gathered at the intersection of the CNTs.
